# Correction to: Epidemiological and clinical characteristics of the first 500 confirmed COVID-19 inpatients in a tertiary infectious disease referral hospital in Manila, Philippines

**DOI:** 10.1186/s41182-021-00346-8

**Published:** 2021-07-13

**Authors:** Kristal An Agrupis, Chris Smith, Shuichi Suzuki, Annavi Marie Villanueva, Koya Ariyoshi, Rontgene Solante, Elizabeth Freda Telan, Kelly Anne Estrada, Ann Celestyn Uichanco, Jocelyn Sagurit, Joy Calayo, Dorcas Umipig, Zita dela Merced, Fe Villarama, Efren Dimaano, Jose Benito Villarama, Edmundo Lopez, Ana Ria Sayo

**Affiliations:** 1 School of Tropical Medicine and Global Health, Nagasaki University, Nagasaki, Japan; 2 San Lazaro Hospital–Nagasaki University Collaborative Research Office, Manila, Philippines; 3 Department of Clinical Research, London School of Hygiene and Tropical Medicine, London, UK; 4 San Lazaro Hospital, Manila, Philippines; 5 Institute of Tropical Medicine, Nagasaki University, Nagasaki, Japan

**Correction to: Trop Med Health 49, 48 (2021)**

**https://doi.org/10.1186/s41182-021-00340-0**

Following publication of the original article [1], a funding information was missing in the Funding section.

The updated Funding section is given below and the changes have been highlighted in **bold typeface**.

**Funding**

This work is in part funded by Nagasaki University (salary support for CS, KAA, and SS). **This study is partially funded by Japan Agency for Medical Research and Development, Grant/Award Numbers: JP19fk0108104h0401, JP20fk0108104h0402.**

The original article [1] has been corrected.
